# Schistosome Syntenin Partially Protects Vaccinated Mice against *Schistosoma mansoni* Infection

**DOI:** 10.1371/journal.pntd.0003107

**Published:** 2014-08-21

**Authors:** Barbara C. Figueiredo, Natan R. G. Assis, Suellen B. Morais, Natasha D. Ricci, Carina S. Pinheiro, Vicente P. Martins, Rodrigo M. Bicalho, Akram A. Da'dara, Patrick J. Skelly, Sergio C. Oliveira

**Affiliations:** 1 Departamento de Bioquímica e Imunologia do Instituto de Ciências Biológicas, Universidade Federal de Minas Gerais, Belo Horizonte, Minas Gerais, Brazil; 2 Instituto Nacional de Ciência e Tecnologia em Doenças Tropicais (INCT-DT), CNPq MCT, Bahia, Brazil; 3 Department of Infectious Disease and Global Health, Cummings School of Veterinary Medicine, Tufts University, North Grafton, Massachusetts, United States of America; 4 Departamento de Biointeração do Instituto de Ciências da Saúde, Universidade Federal da Bahia, Salvador, Bahia, Brazil; 5 Departamento de Biologia Celular do Instituto de Ciências Biológicas, Universidade de Brasília, Brasília, Distrito Federal, Brazil; McGill University, Canada

## Abstract

**Background:**

Schistosomiasis is a neglected tropical disease caused by several species of trematode of the genus *Schistosoma*. The disease affects more than 200 million people in the world and causes up to 280,000 deaths per year, besides having high morbidity due to chronic illness that damages internal organs. Current schistosomiasis control strategies are mainly based on chemotherapy, but many researchers believe that the best long-term strategy to control disease is a combination of drug treatment and immunization with an anti-schistosome vaccine. Among the most promising molecules as vaccine candidates are the proteins present in the tegument and digestive tract of the parasite.

**Methodology/Principal Findings:**

In this study, we describe for the first time *Schistosoma mansoni* syntenin (*Sm*Synt) and we evaluate its potential as a recombinant vaccine. We demonstrate by real-time PCR that syntenin is mainly expressed in intravascular life stages (schistosomula and adult worms) of the parasite life cycle and, by confocal microscopy, we localize it in digestive epithelia in adult worms and schistosomula. Administration of siRNAs targeting *Sm*Synt leads to the knock-down of *syntenin* gene and protein levels, but this has no demonstrable impact on parasite morphology or viability, suggesting that high *SmSynt* gene expression is not essential for the parasites *in vitro*. Mice immunization with r*Sm*Synt, formulated with Freund's adjuvant, induces a Th1-type response, as suggested by the production of IFN-γ and TNF-α by r*Sm*Synt-stimulated cultured splenocytes. The protective effect conferred by vaccination with r*Sm*Synt was demonstrated by 30–37% reduction of worm burden, 38–43% reduction in the number, and 35–37% reduction in the area, of liver granulomas.

**Conclusions/Significance:**

Our report is the first characterization of syntenin in *Schistosoma mansoni* and our data suggest that this protein is a potential candidate for the development of a multi-antigen vaccine to control schistosomiasis.

## Author Summary

Schistosomiasis affects more than 200 million people worldwide and causes up to 280,000 deaths per year. In terms of global mortality and morbidity, this disease is the most important human helminth infection. Current control strategies are based on chemotherapy, but recurrent re-infection of people living in endemic areas makes many researchers, and also the World Health Organization, search for an effective vaccine to provide protection against schistosomiasis. Substantial efforts have been committed to the characterization of new antigens for an anti-schistosome vaccine and, in order to find new targets for vaccine and/or drug development, we searched transcriptomics and proteomics of *Schistosoma mansoni* and identified the protein syntenin (*Sm*Synt) for analysis. In this study, we characterize *Sm*Synt and evaluate its potential as a vaccine candidate to protect mice against *S. mansoni* infection. We demonstrate that *Sm*Synt is expressed in schistosomula and adult worms, the intravascular stages of *S. mansoni* and it is located in the intestinal tract of the worms, an important host/parasite interface. Furthermore, vaccination of mice with r*Sm*Synt confers partial protection against *S. mansoni* challenge infection and ameliorates parasite-induced liver pathology. Our data suggest that *Sm*Synt is a potential candidate in the development of a vaccine against schistosomiasis.

## Introduction

Schistosomiasis, which is caused by trematode worms of the genus *Schistosoma*, is considered the most important human helminth infection in terms of global mortality and occurs primarily in developing countries. In sub-Saharan Africa, higher mortality rates, such as 280,000 deaths per year, have been reported [1], [2]. Around 800 million people live in areas with risk of infection in 76 countries worldwide, of which at least 200 million people are infected [3], [4]. This disease has also high morbidity rates, being responsible for the loss of up to 4.5 million DALYs (disability adjusted life years) annually [3]. Many countries invest in intervention strategies based on short-term control programs using mass drug administration [5], [6]. Conversely, even with massive treatment, disease endemicity has not decreased because poor sanitary conditions lead to constant re-infection [7]–[9]. In addition, the possibility of the emergence of drug resistant parasites has been raised [10], [11]. Many researchers consider that the best strategy for achieving the reduction of disease transmission and morbidity is through immunization with an anti-schistosome vaccine combined with the current drug treatment [12]. A vaccine that induces even a partial reduction in worm burdens could considerably reduce pathology and limit parasite transmission [13].

In order to find new targets for vaccine development, we searched the genomic and transcriptomic data of *Schistosoma mansoni* and identified the protein syntenin for analysis [14], [15]. Syntenin is a scaffold supporting protein that is important in assembling protein complexes involved in the organization of cell-cell and cell-matrix adhesion [16], in cellular trafficking [17], in endocytic recycling of transmembrane receptors [18], in biogenesis of small extracellular vesicles [19] and in exosomes [20], thus playing important roles in cell growth, development and differentiation. Zimmermann and colleagues (2002) characterized the binding of mammalian syntenin-1 with plasma membrane phosphatidylinositol 4,5-bisphosphate (PIP_2_) and showed it to be important for the regulation of the assembly of multiprotein complexes at the cell membrane [21]. Syntenin is also known to play a role in tumor metastasis [22], [23] and in maintaining neuronal synapsis integrity [24], [25].

The functional variety of syntenin binding partners suggests that the protein might have several roles in many essential cellular processes and this hypothesis motivates the present study. Immunological inhibition of schistosome syntenin might block these key functions and debilitate the parasites. Our goal is to characterize *S. mansoni* syntenin (*Sm*Synt) and to test it as a potential vaccine in the murine model of schistosomiasis. We determine that *Sm*Synt is expressed and located on the syncytial surface of the gastrodermis of *S. mansoni* adult worms and schistosomula. Further, we show that high levels of expression of syntenin are not essential for parasite survival *in vitro*. Finally, we find that vaccination with r*Sm*Synt induces a Th1-type of immune response in mice as well as partial reduction in worm burden and liver pathology in vaccinated animals.

## Methods

### Mice and parasites

Six- to eight-week-old female C57BL/6 mice were purchased from the Federal University of Minas Gerais (UFMG) animal facility. Cercariae of *S. mansoni* (LE strain) were routinely obtained from infected *Biomphalaria glabrata* snails at René Rachou Research Center (CPqRR-Fiocruz, Brazil) or at the Molecular Helminthology Laboratory at the Cummings School of Veterinary Medicine (Tufts University, USA) and prepared by exposing infected snails to light for 2 h to induce shedding of parasites. Cercariae numbers and viability were determined using a light microscope prior to infection. Schistosomula were cultivated for at least 7 days *in vitro* after transformation of cercariae, as previously described [26]. For developmental analysis, adult worms were obtained by perfusion of the portal hepatic vein from Swiss Webster mice, 6–7 weeks after infection with approximately 100 cercariae. Parasite eggs were recovered from the livers of these mice.

### Ethics statement

All animal experiments were conducted in accordance with Brazilian Federal Law number 11.794, which regulates the scientific use of animals, and IACUC guidelines. All protocols were approved by the Committee for Ethics in Animal Experimentation (CETEA) at Federal University of Minas Gerais (UFMG) under permit 179/2010 or by the Tufts University Institutional Animal Care and Use Committee under protocol G2012-150.

### Bioinformatic analysis of the schistosome *syntenin* (*SmSynt*) gene

Bioinformatic analyses of the *SmSynt* gene (Smp_068530) were performed as previously described [27]. Briefly, protein sequence of *Sm*Synt was obtained from *Schistosoma* database (http://www.schistodb.org) and used as a query in BLAST searches against the non-redundant protein sequence database to identify syntenin homologs. Alignment of these protein sequences was generated with ClustalX 2.0. The boundaries of the conserved structural domains known as PDZ domains were defined based on ScanProsite online software. Molecular weight (MW) and isoelectric point (pI) were calculated with the Compute pI/Mw tool. Posttranslational modification predictions were conducted with the following software: signal peptide prediction was performed using the SignalP 3.0 server, transmembrane helices were analyzed by TMHMM version 2.0 and SOSUI, O-glycosylation spots were determined by YinOYang, N-glycosylation sites were defined by NetNGlyc 1.0 Server and phosphorylation sites were predicted with NetPhos 2.0 server. For phylogenetic analyses, the evolutionary history was inferred using the Neighbor-Joining method, excluding positions with gaps. The numbers represent the confidence of the branches assigned by bootstrap (in 1000 samplings). All evolutionary analyses were conducted in MEGA6 [28].

### Chemicals

All reagents were purchased from Sigma-Aldrich, CO (St. Louis, MO, USA) unless otherwise specified.

### *SmSynt* gene expression analysis

To monitor *SmSynt* gene expression in schistosome life stages, RNA was extracted from the parasites using the TRIzol method (Invitrogen, Carlsbad, CA, USA) following the manufacturer's instructions. Residual DNA was digested by DNase I from Turbo DNAse kit (Ambion, Inc.) following the manufacturer's instructions. cDNA synthesis was performed using 1 µg RNA, an oligo-dT primer and Superscript reverse transcriptase III (Invitrogen, Carlsbad, CA). For quantitative real-time PCR (qRT-PCR), performed using TaqMan Assays, primer sets and reporter probes were customized and reagents were purchased from Life Technologies (Carlsbad, CA, USA). The following primers and probes were used to detect *SmSynt* gene expression, primers: SmSynt-F, 5′-AAACTCCAATGAAAGTTACTATTATACCAAAGACA -3′; SmSynt-R, 5′-AAGGTAATGATCGATCCATTTGTAATCGT-3′; and SmSynt probe, 5′-FAM-CCACTTGGAAGACTTCT-3′. As an endogenous control, we used the housekeeping *triose phosphase isomerase* (*TPI*) gene, to compare *SmSynt* expression across schistosome life cycle stages, primers: SmTPI-F, 5′-CATACTTGGACATTCTGAGCGTAGA-3′; SmTPI-R, 5′-ACCTTCAGCAAGTGCATGTTGA-3′; and SmTPI probe, 5′-FAM-CAATAAGTTCATCAGATTCAC-3′ or alpha-tubulin for relative quantification following gene knock-down, primers: SmTub-F, 5′-GGTTGACAACGAGGCCATTTATG-3′; SmTub-R, 5′-GCAGTAAACCCTTGGTCAGATAATTTTG-3′; and SmTub probe, 5′-FAM- ATATTTGTCGACGGAAT-3′. Each qRT-PCR reaction was performed using 1 µl of the cDNA, in a final volume of 20 µl. All samples were run in triplicate and underwent 40 amplification cycles on a StepOne Plus system (Life Technologies - Carlsbad, CA, USA). The ΔΔCt method was employed for relative analysis [29]. For graphical representation, ΔΔCt values were normalized to controls and expressed as a percentage difference.

### Preparation and delivery of siRNAs and post RNAi analysis

Two gene-specific small inhibitory RNAs (siRNAs) were commercially synthesized (Integrated DNA Technologies, Inc.) and used to induce *SmSynt* gene expression knock-down. These are siSynt1 and siSynt2. The DNA sequence for siSynt1 is 5′-AACCAGGAGTTAGACTTGTTACTCTTT-3′, spanning coding DNA positions 323–349 and the DNA sequence for siSynt2 is 5′-ACCAACCACTATGCAGTCCAATTGTCA-3′, spanning coding DNA positions 66–92, both designed with the help of the online IDT RNAi Design Tool (https://www.idtdna.com/Scitools/Applications/RNAi/RNAi.aspx). The siRNAs were delivered to 7-day old schistosomula or adult parasites by electroporation as previously described [30]. The control, irrelevant siRNA is: 5′-CT TCCTCTCTTTCTCTCCCTTGTGA-3′. To monitor gene expression at various times post siRNA administration, qRT-PCR was performed using custom TaqMan Assays as described above. To measure protein levels, western blot analysis was performed seven days post siRNA administration as described below. To compare control and siSynt siRNA-electroporated parasite sizes, images were taken using an inverted microscope (TH4–100; Olympus, Tokyo, Japan) equipped with a Retiga 1300 camera (Q Imaging, Surrey, BC, Canada), and the area occupied by individual schistosomula was measured using ImageJ software (U.S. National Institutes of Health, Bethesda, MD, USA). Parasite viability in culture was measured by adding 1 µg/ml Hoechst 33258 to the cultures at room temperature. After 10 min dead parasites (Hoechst positive) were counted microscopically, using a 460 nm reading filter.

### SmSynt protein expression and purification

The plasmid pJ414 containing the complete cDNA sequence for *SmSynt* (pJ414::Synt) was manufactured by DNA 2.0, Inc. USA (https://www.dna20.com) using DNA2.0 optimization algorithms for expression in *Escherichia coli*. This plasmid was transformed into *E. coli* Rosetta-gami (Merck KGaA, Darmstadt, Germany) competent cells. *E. coli* cells harboring the expression plasmid were grown until OD_600_ of 0.5 was reached, then isopropylthiogalactoside (IPTG) was added to a final concentration of 1 mM to induce gene expression. Four hours after induction, the bacteria were harvested by centrifugation and the cell pellet was resuspended in 50 ml of 10 mM Na_2_HPO_4_, 10 mM NaH_2_PO_4_, 0.5 M NaCl and 20 mM imidazole (pH 7.4). Subsequently, the cells were sonicated and centrifuged at 5400 *g* for 20 min. Recombinant *Sm*Synt protein (r*Sm*Synt) was recovered from the pellet fraction that was solubilized in 8M urea and purified by affinity chromatography on a Ni-Sepharose column (HiTrap chelating 5 mL) using an AKTA explorer chromatography system, following the manufacturer's instructions (GE Healthcare, São Paulo, Brazil). Fractions containing the protein, identified by SDS/PAGE-12% were dialyzed against PBS pH 7.0 at 4°C using Spectra/Por2 membrane (MWCO 6 to 8 kDa; Spectrum Medical Industries, Inc., Laguna Hills, CA). The recombinant protein was quantified using the Bradford method [31] and used as antigen for vaccination and immunological experiments.

### Mice immunization, challenge infection and worm recovery

Two groups of 10 mice each (female C57BL/6, aged 6–8 weeks) were subcutaneously injected with 25 µg of r*Sm*Synt or PBS (negative control) on days 7, 22 and 37. Both preparations were formulated with Complete Freund's Adjuvant (CFA) for the first immunization and Incomplete Freund's Adjuvant (IFA) for the following two. Fifteen days after the last immunization, mice were challenged through percutaneous exposure of abdominal skin to water containing 100 cercariae (LE strain) for 1 h. Forty-five days after challenge, adult worms were perfused from the portal veins of each animal and the vaccine protection level was calculated, as previously described [32]. Two independent experiments were performed and, in the second vaccination trial, c*Sm*AQP was also used as a negative control, since vaccination with this protein has been shown not to lead to any worm burden reduction or pathology reduction in mice following challenge infection with *S. mansoni* [33].

### Measurement of specific anti-rSmSynt antibodies

Following immunization, sera were collected from mice in each experimental group at two week intervals. The levels of specific anti-r*Sm*Synt antibodies were measured by indirect ELISA, as previously described [15]. Briefly, microtiter plates (Nunc, Roskilde, Denmark) were coated with 5 µg/mL r*Sm*Synt and polyclonal anti-*Sm*Synt antibodies were detected using peroxidase-conjugated anti-mouse IgG, IgG1 or IgG2a (Southern Biotechnology, CA, USA), following the manufacturer's instructions. Color reaction was induced by adding 100 µL of 200 pmol OPD (o-phenylenediamine) in citrate buffer (pH 5.0) plus 0.04% H_2_O_2_ to each well for 10 min. The color reaction was stopped by adding 50 µL of 5% sulfuric acid to each well. Plates were read at 495 nm in an ELISA plate reader (BioRad, Hercules, CA, USA).

For subsequent western blot and immunolocalization experiments, anti-r*Sm*Synt antibodies were affinity purified. To perform this, 500 µg of the recombinant protein was first adsorbed to a nitrocellulose membrane. The membrane was then blocked with TBST (tris-buffered saline, pH 7.2 with 0.05% Tween-20) containing 5% non-fat dry milk. After washing with TBST, the membrane was incubated with 2 mL of pooled serum samples from the fourth bleed (day 45 - after the third immunization and before challenge) for 16 h at 4°C. After another set of washings with TBST, bound antibodies were eluted with 400 µl 0.014% triethylamine for 5 min at room temperature and then neutralized by the addition of 100 µL 10× TBS. This material was dialyzed against TBS pH 7.0 at 4°C using a Spectra/Por2 membrane (MWCO 6 to 8 kDa; Spectrum Medical Industries, Inc., Laguna Hills, CA).

### Cytokine analysis

Cytokine measurement experiments were performed using cultured splenocytes from individual PBS- or r*Sm*Synt-vaccinated mice (n = 4). Splenocytes were obtained from the macerated spleens of individual mice one week after the third immunization or 45 days after the challenge. Spleen cells were maintained in culture with medium alone or were stimulated with r*Sm*Synt protein (25 µg/mL). Positive control conditions included stimulation with concanavalin A (ConA) (5 µg/mL), or LPS (1 µg/mL), as previously described [32]. The culture supernatants were collected after 24 h to measure IL-5 and IL-4 levels, after 48 h to measure TNF-α levels and after 72 h to measure IFN-γ and IL-10 levels. Cytokine measurement assays were performed using the DuoSet ELISA kit (R&D Systems, Minneapolis, MN) according to the manufacturer's instructions.

### Immunolocalization of SmSynt in *S. mansoni* adult worms and schistosomula

To immunolocalize *Sm*Synt, adult worms were first recovered from perfused mice, and schistosomula were prepared *in vitro* as described [26]. Parasites were next fixed in Omnifix II (Ancon Genetics, St Petersburg, FL, USA). For sectioning assays, 7 µm slices of paraffin-embedded adult parasites were deparaffinized using xylol and hydrated with an ethanol series [34]. For experiments using *in vitro* 7-day cultured schistosomula, a whole-mount protocol was used. Schistosomula were treated with permeabilizing solution (0.1% Triton X-100, 1% BSA and 0.1% sodium azide in PBS pH 7.2) overnight at 4°C [34]. Next, schistosomula and parasite sections were blocked with 1% BSA (bovine serum albumin) in PBST (phosphate buffered saline, pH 7.2 with 0.05% Tween-20) for 1 h and incubated with anti-r*Sm*Synt serum diluted 1∶20 in blocking buffer. Serum from non-immunized mice was used as a negative control. The samples were washed with PBST and incubated with an anti-mouse IgG antibody conjugated to FITC (Molecular Probes, Carlsbad, CA, USA) diluted 1∶100 in blocking buffer containing rhodamine phalloidin 1∶100 (Molecular Probes, Carlsbad, CA, USA) to stain actin microfilaments. Samples were washed and mounted with ProLong Gold anti-fading mounting medium containing DAPI (Molecular Probes, Carlsbad, CA, USA). Samples were imaged on a Nikon Eclipse Ti fluorescence microscope from the Microscopy Center of the Biological Sciences Institute (CEMEL) at the Federal University of Minas Gerais (UFMG).

### Egg count and histopathological analysis

To evaluate the effect of our vaccine on liver pathology, liver tissues from eight mice per group were collected following perfusion. Liver fragments from each animal were weighted and digested with 10% KOH. Released eggs were obtained by centrifugation at 900 *g* for 10 min and resuspended in 1 mL saline. Egg number was counted using a light microscope and the number of eggs recovered per gram of liver tissue was calculated. Liver samples removed from the central part of the left lateral lobe were fixed with 10% buffered formaldehyde in PBS. Histological 6 µm sections were obtained and stained on a slide with haematoxylin-eosin (HE). The number of granulomas was counted using a light microscope with 10× objective lens. Each liver section was scanned to calculate its total area (mm^2^) using ImageJ software (U.S. National Institutes of Health, Bethesda, MD, USA). To measure the total area of granulomas, a microscope with 10× objective lens was used; images were obtained using a JVC TK-1270/RBG microcamera attached to the microscope. Twenty granulomas, each with a single, well-defined egg, were randomly selected in each liver section and the granuloma area was measured using ImageJ software (http://rsbweb.nih.gov/ij/index.html).

### SDS-PAGE and western blot analysis

Purified recombinant protein was analyzed on 15% polyacrylamide SDS-PAGE gels prepared and run as previously described [35]. Proteins were transferred to a nitrocellulose membrane [36] that was blocked with TBST (tris-buffered saline pH 7.5, 0.05% Tween 20) containing 5% dry non-fat milk powder. The membrane was then incubated either with a mouse monoclonal antibody to the 6×His-tag (GE Healthcare, Pittsburgh, PA, USA) fused to rSmSynt or with polyclonal antibodies to r*Sm*Synt (diluted 1∶100) for 1 h at room temperature. After washing with TBST, the membrane was incubated with goat anti-mouse IgG conjugated to alkaline phosphatase (GE Healthcare, Pittsburgh, PA, USA) according to the manufacturer's instructions, then treated with a developing buffer containing nitroblue tetrazolium (NBT) and 5-bromo-4-chloro-3-indolyl-1-phosphate (BCIP).

### Statistical analysis

For qRT-PCR data, one way analysis of variance (ANOVA) and Tukey as the post hoc test was used. Cytokine and antibody analyses were performed using two-way ANOVA and Bonferroni adjustments were included for comparisons between groups. The post-vaccination data (worm burden, egg count, and histopathology) were compared by paired *t*-test. The *p*-values obtained were considered significant if they were <0.01. Statistical analyses were performed using GraphPad Prism 6 (La Jolla, CA).

### Accession numbers

*S. mansoni* syntenin (XP_002578073.1); *S. mansoni* aquaporin (ACI31185.1); *H. sapiens* syntenin (NM_001007067.1); *M. musculus* syntenin (NP_001091697); *C. sinensis* syntenin (GAA48733.1); *S. japonicum* syntenin (AAX27791.2)

## Results

### *Schistosoma mansoni* syntenin

**Figure 1:**
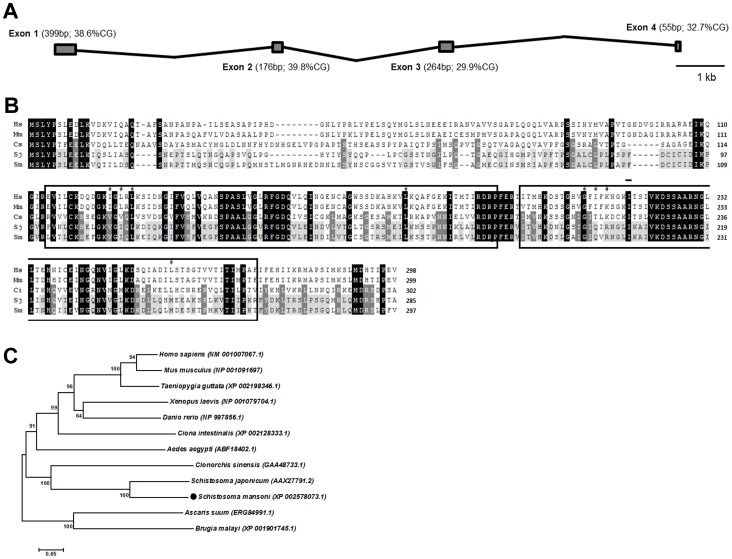
Bioinformatic analysis of *Schistosoma mansoni* syntenin (SmSynt). (A) Schematic representation of the *S. mansoni syntenin* gene. The *SmSynt* gene (Smp_068530) has 4 exons, indicated by grey rectangles, the length of each exon and its GC content are indicated in parentheses. (B) Alignment of the protein sequences of syntenin homologs from human, mouse and trematode species using ClustalX 2.0. Black background indicates identity in all variants, dark grey indicates identity in trematodes, light grey indicates identity in schistosomes, white background indicates a non-conservative residue, dashes indicate gaps introduced to facilitate alignment. The two conserved PDZ domains are contained within black boxes as defined by ScanProsite online software. (#) indicates the residues forming the hydrophobic pockets of PDZ domains [19]. GenBank Accession Numbers: Hs, *Homo sapiens* (NM_001007067.1); Mm, *Mus musculus* (NP_001091697); Cs, *Clonorchis sinensis* (GAA48733.1); Sj, *Schistosoma japonicum* (AAX27791.2) and Sm, *Schistosoma mansoni* (XP_002578073.1). (C) Phylogenetic tree of *Sm*Synt and homologs from other species generated using the Neighbor-Joining method. Bootstrap (1000 replicates) percentages are indicated at each branch. The tree is drawn to scale with branch lengths in nucleotide substitutions per site, the same units used to infer the phylogenetic tree. Analyses were conducted in MEGA6. GenBank accession numbers are given in parentheses.

In a previous study from our lab, syntenin was identified as a down-regulated gene in worms recovered from mice immunized with a recombinant schistosome tegumental protein *Sm*29 (r*Sm*29) [15]. According to the *S. mansoni* genome database (http://www.schistodb.org) the *syntenin* gene is located on chromosome 6. The *SmSynt* gene is around 15 kb in size and consists of 4 exons, sized 399 bp, 176 bp, 264 bp and 55 bp, all of them with similar (∼30–40%) GC content (Figure 1A). The *Sm*Synt cDNA potentially encodes a 297-aa protein, predicted as a soluble 32-kDa protein with a pI of 8.8. Analysis with ProScan online software revealed that the *Sm*Synt sequence has two predicted adjacent conserved structural domains termed PDZ domains, comprising residues 113–192 and 197–272. Four phosphorylation sites were predicted in the second PDZ domain, at S^204^, S^225^ and T^199^ and T^262^. Phosphorylation of one or more of these amino acid residues might change *Sm*Synt interactions with other proteins, changing the way it regulates cellular processes. Three additional residues at the N-terminal region might also be phosphorylated, S^6^, S^20^ and S^89^; and two asparagine residues are predicted to be N-glycosylated, N^38^ and N^50^. No signal peptide or O-glycosylation sites were predicted.

ClustalX 2.0 alignment of *Sm*Synt sequence with syntenin homologs from the trematodes *S. japonicum* and *Clonorchis sinensis* and from human and mouse reveal that *Sm*Synt displays 74% identity with its homologue from *S. japonicum* and 43% identity with the *C. sinensis* homologue (Figure 1B). The sequences of syntenin homologs from several taxa were used to create a phylogenetic tree using the Neighbor-Joining method (Figure 1C) using the MEGA software [28]. *Sm*Synt clusters with *S. japonicum* and *C. sinensis* homologs to form a trematode clade, divergent from the other groups examined. Additionally, the parasitic nematode (*A. suum* and *B. malayi*) syntenin homologs grouped together in a separate clade.

### *SmSynt* is highly expressed in intravascular life stages

**Figure 2:**
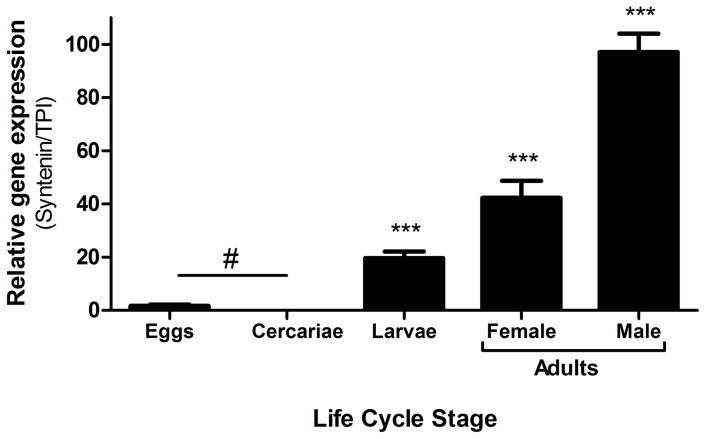
Expression profile of *SmSynt* at different stages in the *S. mansoni* life cycle. Quantitative RT-PCR data showing relative expression level (mean +/− SD) of *Sm*Synt at different stages in the *S. mansoni* life cycle: eggs, cercariae, schistosomula (7-day cultured larvae) and adult worms, female and male (set at 100%). Results are representative of two independent experiments. No significant difference is represented as #. Significant differences from eggs/cercariae and the intravascular phases are denoted by ***, p<0.001.

The expression of *SmSynt* was evaluated in different stages in the life cycle of *S. mansoni* (Figure 2). The *SmSynt* gene exhibits higher relative expression in the intravascular life stages. We detected very low *SmSynt* expression levels in eggs and in cercariae. *Sm*Synt expression increases in the 7-day old schistosomula larval stage (to 20% of the adult male value) and its highest levels are found in adult parasites, with the male worm expressing approximately two times more *Sm*Synt than the female. These observations suggest that *Sm*Synt might be more important in the intravascular life of the parasite (Figure 2).

### Suppression of *SmSynt* gene expression does not affect *S. mansoni*

**Figure 3:**
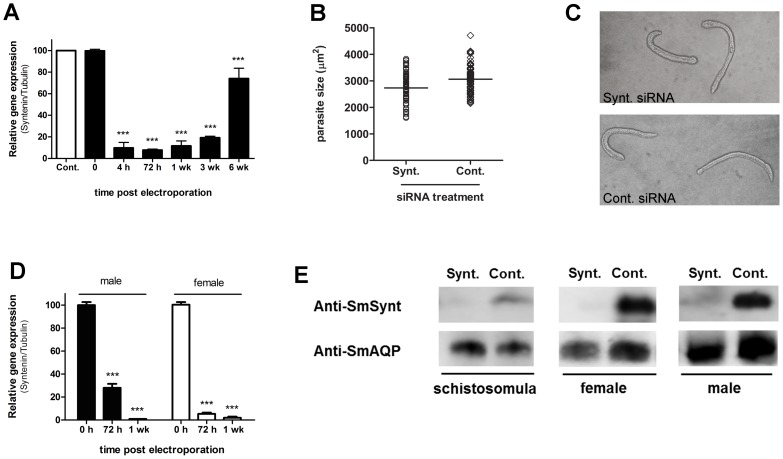
Syntenin gene suppression using RNA interference. (A) *SmSynt* gene expression in cultured schistosomula at different time points after treatment with syntenin siRNAs. Black bars represent the relative expression of the group treated with syntenin siRNAs compared to the group treated with a control siRNA (set as 100%, white bar) in the same time points. Results shown are representative of two replicate experiments. Significant differences from time 0 compared to other time points are denoted by *** for p<0.001. (B) Sizes of schistosomula cultured for three weeks post siRNA treatment. Sizes of individual schistosomula treated with Syntenin (Synt.) or Control (Cont.) siRNAs are shown. The lines indicate the means for each group. Results shown are representative of two replicate experiments. No significant difference was observed between the groups. (C) Representative images of schistosomula treated with either Syntenin (Synt. - upper panel) or Control (Cont. – lower panel) siRNAs, three weeks after siRNA treatment. (D) *Sm*Synt gene expression in cultured adult schistosome male (black bars) or female (white bars) at different time points after treatment with syntenin siRNAs. Bars represent the relative expression of the group treated with Synt siRNAs compared to the group treated with control siRNA (100%) at the same time points. Results shown are representative of two replicate experiments. Significant differences from time 0 and the other time points are denoted by *** for p<0.001 (E) Detection by western blot of *Sm*Synt protein (top row), and a control protein – schistosome aquaporin (*Sm*AQP, bottom row) in extracts prepared from parasites after treatment with syntenin (Synt.) or control (Cont.) siRNAs. Schistosomula were prepared 7 days after treatment and adult parasites were prepared 72 h after electroporation. Diminished levels of *Sm*Synt protein is seen in the first lane of each group of samples. Western blot analysis detecting the control *Sm*AQP protein shows roughly equivalent protein amounts per lane. Results shown are representative of two replicate experiments.

The expression of *SmSynt* was suppressed *in vitro* in schistosomula, and also in male and female adult worms. Figure 3A shows that in schistosomula, 4 hours after siRNA treatment, transcriptional suppression of *SmSynt* was close to 90% when compared to the control group. Suppression remained at over 80% up to at least three weeks post siRNA treatment. After 6 weeks, the suppressive effect waned and *SmSynt* gene suppression was only ∼20% less than controls. At all-time points, suppressed parasites exhibited no overt difference in phenotype compared to controls. Three weeks after siRNA administration, when *SmSynt* gene suppression was about 80%, we measured parasite size. Schistosomula that had their *SmSynt* gene knocked-down showed no significant difference compared to controls in all parameters evaluated: size (Fig. 3B), morphology (Figure 3C shows representative viable worms) and relative viability - 63.1±1.6% and 61.2±5.8% for treatment with Synt and control siRNA, respectively. Suppression of *SmSynt* gene expression in adult parasites was >95% after one week (Fig. 3D), but no significant differences in morphology, viability or behavior were observed between *Sm*Synt-suppressed versus control parasites during three weeks in culture (data not shown).The suppression of the *SmSynt* gene also resulted in a reduction in *Sm*Synt protein production as shown by Western blot analysis using anti-r*Sm*Synt polyclonal antibodies (Fig. 3E). In the case of schistosomula (Figure 3E, left panel), female (Figure 3E, center panel) or male (Figure 3E, right panel), western blot analysis reveals lower *Sm*Synt detection in extracts of parasites treated with siRNA targeting syntenin (Synt.) compared to parasites treated with a control irrelevant siRNA (Cont.). All western blots were stripped and probed with an anti *S mansoni* aquaporin antibody (anti-*Sm*AQP) (Figure 3E) to show that all lanes contain roughly equivalent amounts of protein [37]. Since robust *SmSynt* gene suppression did not measurably impact schistosomes, we conclude that high level expression of this protein is not essential for parasite development and survival in culture.

### Heterologous expression and purification of recombinant SmSynt (rSmSynt)

**Figure 4:**
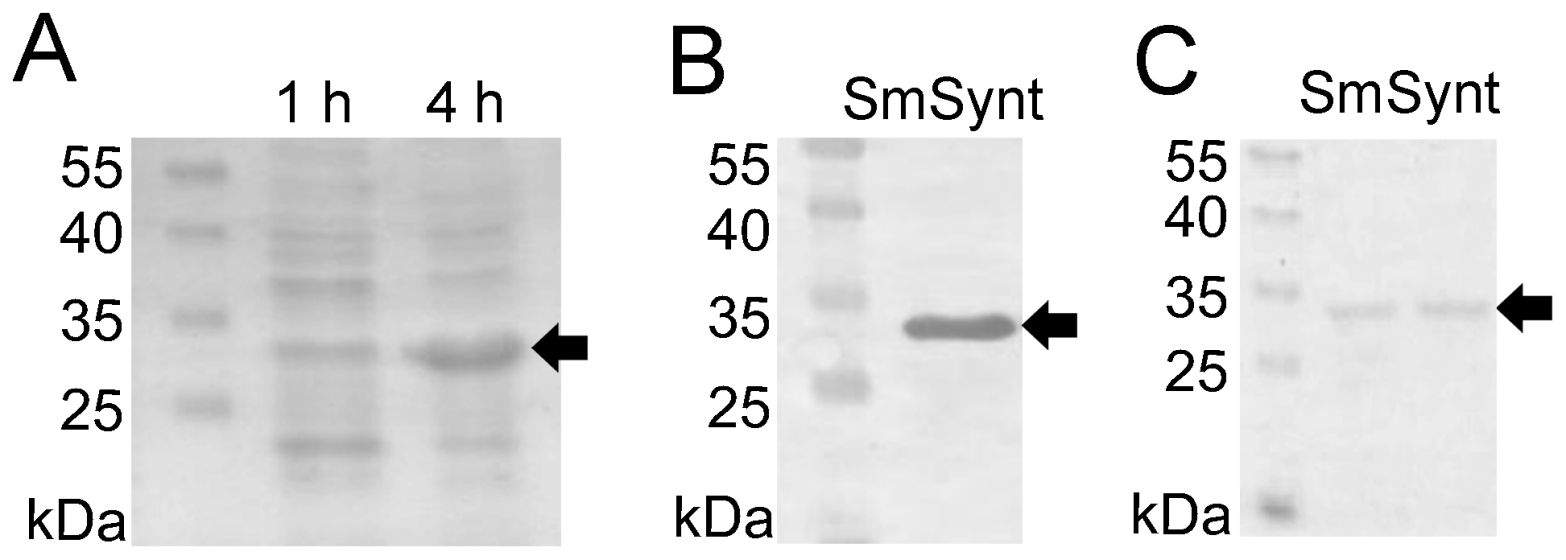
Heterologous expression and purification of r*Sm*Synt. (A) Lysates of *E. coli* expressing r*Sm*Synt were obtained either 1 hour (1 h) or 4 hours (4 h) after IPTG induction and were resolved by SDS-PAGE and stained with Coomassie brilliant blue. A prominent ∼32 kDa protein (*Sm*Synt) is detected in the 4 h lane (arrow). (B) Western blot analysis of the *E. coli* lysate, 4 hours after IPTG induction, probed with monoclonal mouse anti-His tag antibodies. A single ∼32 kDa protein (*Sm*Synt) is detected (arrow). (C) SDS-PAGE gel stained with Coomassie brilliant blue showing two eluted and dialyzed samples of r*Sm*Synt after purification by Ni^2+^-charged column chromatography. Ten microliters were loaded per lane. The left lanes in all cases show molecular mass markers whose sizes are indicated (kDa).

The *SmSynt* coding DNA was cloned into the pJexpress plasmid as described above. Heterologous expression was induced in *E. coli* and bacterial extracts were analyzed by SDS-PAGE followed by Coomassie blue staining. A prominent band of the approximate expected size of r*Sm*Synt (∼32 kDa) was observed in the induced cells (Figure 4A, 4 h time point, arrow). Bacterial extracts were subjected to western blot analysis using a mouse monoclonal anti-His tag antibody. This analysis confirmed that the induced protein of around 32 kDa had a histidine tag (Figure 4B, arrow). The histidine-tagged r*Sm*Synt protein was purified from bacteria lysates under denaturing conditions using nickel affinity chromatography (Figure 4C) and was used for vaccination and immunological studies.

### SmSynt is localized in the syncytium of the intestinal tract of adult *S. mansoni*

**Figure 5:**
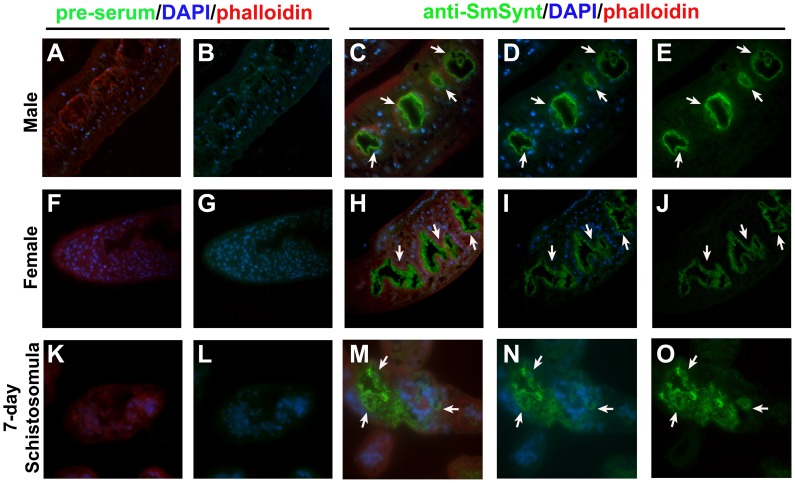
Immunolocalization of *Sm*Synt in *S. mansoni* adult worms and schistosomula. Mouse purified, polyclonal anti-r*Sm*Synt and anti-mouse secondary antibodies conjugated with Alexa 488 (green) were used to label the native *Sm*Synt protein in sections of 7-day schistosomula and adult worms (C–E, H–J, M–O). As a control, serum from naive mice was tested in a similar manner (A–B, F–G, K–L). Nuclei stained with DAPI (blue), actin stained with phalloidin conjugated to rhodamine (red). White arrows indicate the localization of *Sm*Synt on the surface and lumen of the intestinal lining of adult worms and schistosomula.

In order to determine the localization of *Sm*Synt in *S. mansoni* adult parasites (male and female) and 7-day cultured schistosomula, parasites were stained with polyclonal antibodies raised against r*Sm*Synt. *Sm*Synt (green staining, arrows Figure 5) was located mainly in the syncytium of the gut in the life cycle stages analyzed (Fig. 5C–E, H–J and M–O). Sera from naïve mice (pre-serum, Figure 5), used as a negative control, did not detect any fluorescent signal in the gut or elsewhere in either the adult parasites (Fig. 5A, B, F and G) or schistosomula (Fig. 5K and L). The cell nuclei were stained with DAPI (blue) and rhodamine phalloidin (red) was used to stain actin in the cytoskeletal tegument components and muscle layers.

### rSmSynt vaccination and antibody profile

**Figure 6:**
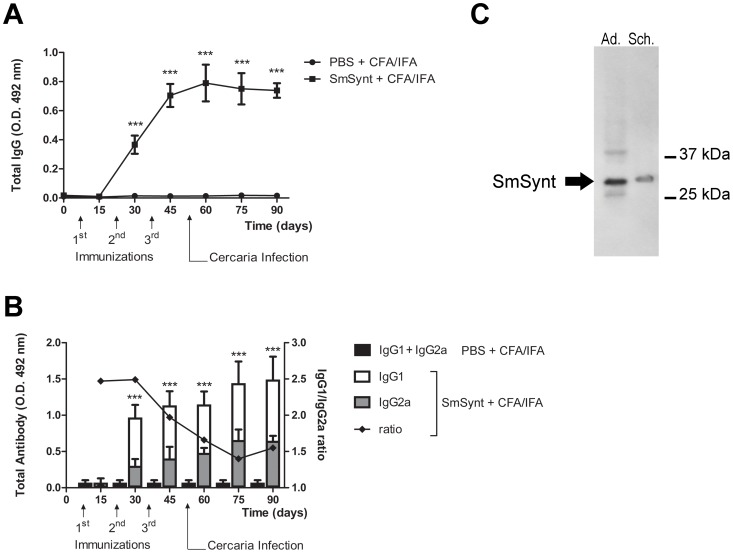
Humoral immune response to r*Sm*Synt vaccination. (A) Sera from r*Sm*Synt-vaccinated (black squares) and control (black circles) mice (mean +/− SD, n = 10) were tested by ELISA to evaluate the levels of specific IgG antibodies to r*Sm*Synt at the indicated time points during the vaccination trial. The vaccination scheme is depicted below the x-axis. Significantly greater levels of anti-r*Sm*Synt IgG antibodies were detected in the r*Sm*Synt-immunized group compared to controls, from the second immunization onwards. (B) IgG isotypes IgG1 and IgG2a levels (mean +/− SD, n = 10) detected in r*Sm*Synt-immunized versus control mice at the indicated time points during the vaccination trial. The vaccination scheme is depicted below the x-axis. The IgG1/IgG2a ratio is given (black diamonds). (C) Ten micrograms of protein extracts from adult parasites (Ad.) and schistosomula (Sch.) were resolved by SDS-PAGE, blotted to nitrocellulose membrane and probed with purified, polyclonal anti-r*Sm*Synt antibodies. The antisera detect a dominant band with the predicted size of syntenin, ∼32 kDa in both extracts (arrow); two minor protein bands (likely non-specific) are also seen in the adult lane. As a control, serum from naïve mice does not appreciably stain the membrane (not shown).

Recombinant syntenin (r*Sm*Synt) was produced and tested as a vaccine candidate against schistosomiasis. C57BL/6 mice were immunized with 3 doses of the vaccine formulated as 25 µg of r*Sm*Synt (or PBS as a control) in Freund's adjuvant (CFA/IFA). After immunization, mice were challenged with 100 cercariae as described earlier. Sera from 8–10 animals from each group were tested by ELISA to evaluate the levels of specific IgG or IgG subclasses (IgG1 and IgG2a) antibodies to r*Sm*Synt. Robust titers of anti-r*Sm*Synt IgG antibodies were detected at all-time points after the second immunization (Figure 6A). From day 30 onwards, mice immunized with r*Sm*Synt produced significantly higher levels of anti-r*Sm*Synt IgG antibodies when compared to PBS-immunized animals (p<0.01). The measurement of IgG isotypes revealed that immunization with r*Sm*Synt induced the production of anti-r*Sm*Synt IgG1 and IgG2a after the second injection (Figure 6B). Furthermore, a reduced IgG1/IgG2 ratio was observed after the third immunization and this suggests the development of a Th1 immune response induced by vaccination with r*Sm*Synt formulated with Freund's adjuvant. Additionally, western blot analysis was performed to determine whether polyclonal antibodies raised against r*Sm*Synt following vaccination were able to recognize the native protein in schistosome extracts. A major protein of the expected size of *Sm*Synt (∼32 kDa) was detected in adult worms and schistosomula extracts (Figure 6C). Sera from naïve mice were used as a control and these did not appreciably detect schistosome proteins (data not shown). This result indicates that the anti-r*Sm*Synt antibody was able to recognize native *S. mansoni* syntenin.

### Cytokine profile after rSmSynt vaccination

**Figure 7:**
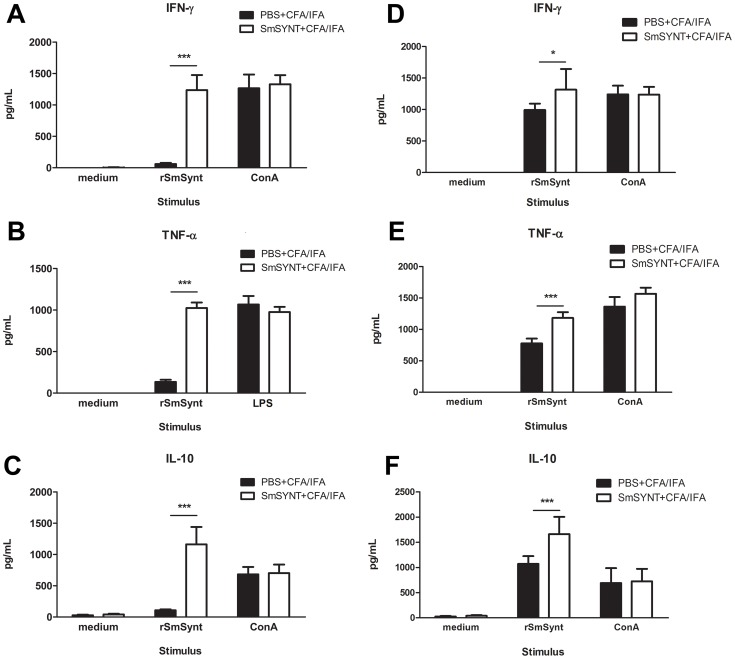
Cytokine profile of splenocytes recovered from r*Sm*Synt-vaccinated versus control mice. Ten days after the final immunization with r*Sm*Synt or PBS, splenocytes from five mice were isolated and assayed for their IFN-γ (**A**), TNF-α (**B**) and IL-10 (**C**) production (mean +/− SD) in response to stimulation with r*Sm*Synt (25 µg, middle group) or ConA/LPS (right group) or medium (left group). The cytokine profile was also evaluated after the challenge with cercariae where high levels of IFN-γ (**D**), TNF-α (**E**) and IL-10 (**F**) were detected as well. Significant differences between data from mice immunized with r*Sm*Synt and the PBS-control group are denoted by *, p<0.05 or ***, p<0.001.

Following vaccination, we measured the production of IFN-γ, TNF-α, IL-4, IL-5 and IL-10 in supernatants of spleen cells stimulated with r*Sm*Synt. We detected significantly higher levels of IFN-γ (1235±240 pg/ml) and TNF-α (1024±134 pg/ml) in spleen cells from animals immunized with r*Sm*Synt when compared to the PBS-immunized control group (Figures 7A–B, r*Sm*Synt stimulus group). Th2 cytokines, IL-4 and IL-5, were produced in very low levels with no statistically significant differences detected between vaccinated and control groups (data not shown). Significantly higher levels of the modulatory cytokine IL-10 (1161±278 pg/ml) were also observed in r*Sm*Synt-vaccinated animals compared to controls (Figure 7C).

These results indicate that immunization with r*Sm*Synt formulated with Freund's adjuvant induces a Th1 immune response characterized by the production of high levels of the Th1 cytokines IFN-γ and TNF-α and the absence of the Th2 cytokines, IL-4 and IL-5.

Moreover, we evaluated the cytokine profile after challenge with cercariae. Similarly to previous analysis, Th1 cytokines IFN-γ (1315±2329 pg/ml) and TNF-α (1182±204 pg/ml) were detected in the supernatant of spleen cells from animals immunized with r*Sm*Synt (Figures 7D–E, r*Sm*Synt stimulus group). Neither IL-4 nor IL-5 was detected and high levels of IL-10 (1661±341 pg/ml) were maintained after infection.

Concanavalin A (ConA) or LPS were used as positive controls to confirm that splenocytes were responsive to stimuli (Figure 7, ConA/LPS group). Spleen cells stimulated with medium alone produced no, or negligible, cytokines (Figure 7, medium group).

### Immunization with rSmSynt induces partial protection in mice

**Figure 8:**
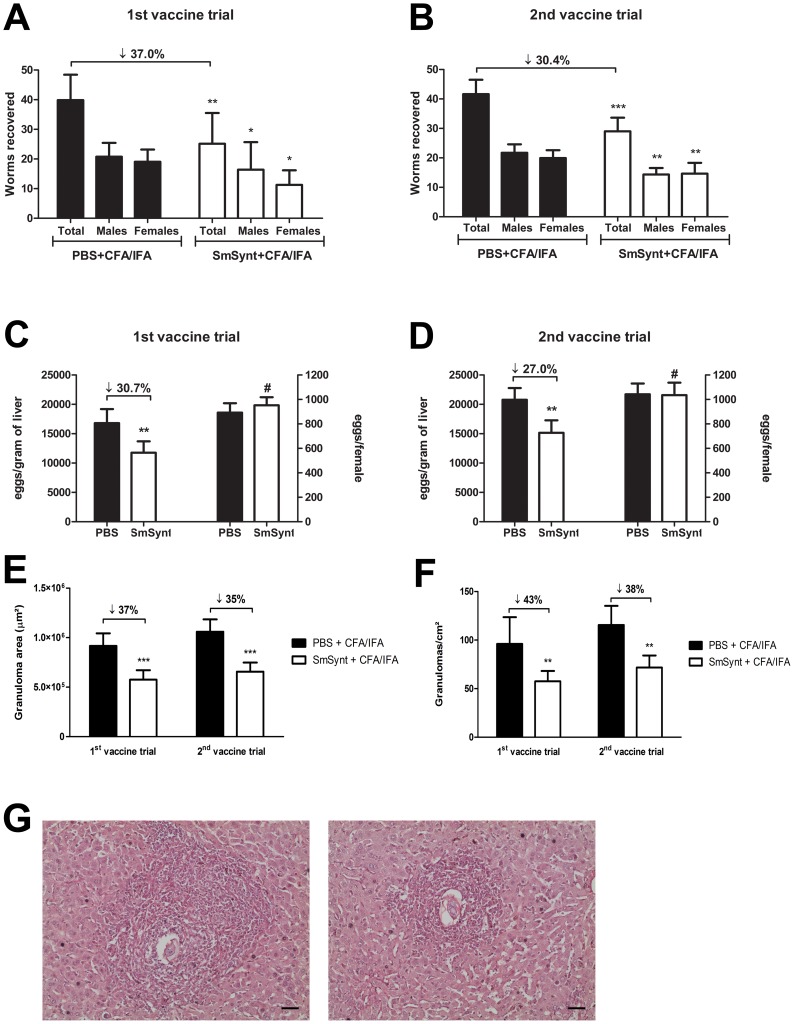
Vaccination with r*Sm*Synt reduces the worm burden and liver pathology in mice. Worm burden (mean +/− SD) in control mice injected with PBS versus mice immunized with r*Sm*Synt and challenged with *S. mansoni* cercariae. A and B show data from two independent vaccine trials. Mice vaccinated with r*Sm*Synt showed a 37% reduction in the adult worm burden in the first trial and a 30.4% reduction in the second trial. (C,D) Left bars: Number of eggs per gram of liver tissue (mean +/− SD) in the first and second trials, respectively. Right bars: ratio of number of eggs to number of recovered females, showing that egg reduction is proportional to worm reduction. (E) Granuloma area (mean +/− SD) in liver sections of r*Sm*Synt-vaccinated (while bars) versus control (black bars) mice. A 37% reduction is seen in trial 1 (left) and a 35% reduction is seen in trial 2 (right). (F) Granuloma number (mean +/−SD) in liver sections of r*Sm*Synt-vaccinated (white bars) versus control (black bars) mice. A 43% reduction in the number of granulomas in vaccinated mice is seen in vaccine trial 1 (left) and a 38% reduction is seen in trial 2 (right). (G) Representative image of a granuloma detected in a hematoxylin and eosin-stained liver section from a PBS-injected control mouse, (left) compared to that from a mouse vaccinated with r*Sm*Synt, (right). The bar represents 0.05 mm. Asterisks indicate statistically significant differences between the vaccinated groups and the control group (* *p*<0.01, ** *p*<0.05 and *** *p*<0.001) and # indicates no significant difference between groups.

Two independent vaccination trials were conducted in C57BL/6 mice. The animals were immunized three times with r*Sm*Synt or PBS as a control, both formulated with Freund's adjuvant and then, protective immunity was evaluated 45 days after challenge with 100 *S. mansoni* cercariae. Mice vaccinated with r*Sm*Synt showed a 37% reduction in adult worm burden in the first trial (Fig. 8A) and a 30.4% reduction in the second trial (Fig. 8B). We also observed reductions in the number of eggs in mouse livers. The number of eggs per gram of liver tissue was reduced 30.7% and 27.0% in in the first and second trials, respectively (Fig. 8C and D – left bars). However, this reduction was proportional to the female worm burden reduction (Fig. 8C and D – right bars), which suggests that the vaccination probably did not affect the ability of the females to produce and release eggs. Additionally, analysis of the hepatic tissue demonstrated that r*Sm*Synt immunization reduced liver pathology. Histopathological analysis showed a 35–37% reduction in granuloma area (Fig. 8E), and 38–43% reduction in liver granuloma counts (Fig. 8F), as compared to control mice. Figure 8G shows a representative image of a granuloma from a liver section of a PBS control mouse (on the left) and a r*Sm*Synt-vaccinated mouse (on the right). As negative control for protection assays, we also used on the second vaccination trial the schistosome aquaporin with Freund's adjuvant that we have shown previously to not induce protective immunity. The worm burden observed in aquaporin vaccinated mice was similar to PBS/CFA control group (data not shown).

## Discussion

Searching for new vaccine targets, our group has tested many *Schistosoma mansoni* antigens [38]–[42], including the tegument protein *Sm*29 which conferred up 50% protection in mice [15]. We showed that worms recovered from mice immunized with r*Sm*29 had a reduced expression of almost 500 genes, among them, some surface antigens, such as *Sm*23 [15]. Reduced expression of these genes may impair parasite adaptation to the host in the face of an ongoing anti-parasite immune response in the *Sm*29-vaccinated animals. Herein, we examine one of these down-regulated genes, syntenin. Up to now, nothing is known about the function of syntenin in schistosomes and other parasitic helminths. However, in other systems, syntenins are important scaffold proteins, involved in many essential cellular processes such as vesicle transportation [17] and cell-cell adhesion [16]. In this study, we clone and characterize schistosome syntenin (*Sm*Synt) and test its vaccine potential.

Phylogenetic analysis demonstrates that *Sm*Synt forms, together with its homologs from *S. japonicum* and *Clonorchis sinensis*, a clade containing only trematode syntenins, divergent from other eukaryotic groups. *Sm*Synt displays low identity to human and mouse homologs (39% for both) and higher identity to *S. japonicum* syntenin (74%). Syntenins from all analyzed species contain two conserved PDZ domains in tandem, connected by a four-amino acid linker that is also highly conserved. The PDZ domain is a widespread protein-protein interaction component that is found in bacteria, fungi, and metazoans [43]. PDZ domain–containing proteins are involved in the control of diverse and essential physiologic processes, depending on their binding partners [44]. Likely *Sm*Synt also binds to partner proteins via its PDZ domains but, as yet, no *Sm*Synt-binding protein has been identified.

The expression of *Sm*Synt is higher in the schistosome intravascular life stages, which involves infection and disease development in the human host. We carried out RNA interference studies in schistosomula and adult worms to knock-down *Sm*Synt transcript levels and then, we observed the development and behavior of these worms in culture for several weeks. We observed up to 95% knock-down of *syntenin* gene expression and a marked reduction in *Sm*Synt protein levels in all parasite stages tested; nevertheless, following gene knock-down we observed no evident phenotypic alteration in the life stages analyzed. This demonstrates that high level *Sm*Synt expression is not essential for schistosome survival *in vitro*. We hypothesize that robust syntenin expression *in vivo* is crucial for the parasites. Previous studies of syntenin knock-down in other systems revealed interesting phenotypes: in zebrafish, the depletion of syntenin-a in the whole embryo impairs body-axis formation [45]; in tumoral lineages, the absence of syntenin diminishes cell migration and metastatic potential [46] and in neurons, syntenin knock-down promotes an increase in the numbers of neurites and branches [47]. Further studies are required to define the function and requirement of schistosome syntenin *in vivo*.

The immunolocalization experiments using polyclonal anti-r*Sm*Synt indicated that *Sm*Synt is mainly present in the intestinal tract in adult parasites, and to the internal tissues of larval stage schistosomula. The schistosome gastrodermis consists of a syncytial epithelium with many vacuolar compartments, irregular villi and numerous lamellae that project into the lumen [33], [48]. Likely *Sm*Synt plays a role in cellular trafficking in this tissue [17]. Since the schistosome gastrodermis is essential for blood feeding and is accessible to host macromolecules such as albumin and immunoglobulins [21], [29], we decided to test *Sm*Synt as an immunogen in the development of an anti-schistosome vaccine. We designed a plasmid with a synthetic *Sm*Synt cDNA with optimized codon usage that allowed us to produce the recombinant protein efficiently in *E. coli*. Recombinant *Sm*Synt was purified and used in the formulation of our vaccine, together with Freund's adjuvant. Immunization with this formulation induced high levels of anti-r*Sm*Synt IgG production after the second vaccination. The ratio between the IgG subtypes (IgG1/IgG2a) decreased after the third immunization, which suggests a shift to Th1 immune response. Confirming this hypothesis, cytokine analysis of the supernatants of cultured splenocytes stimulated with r*Sm*Synt yielded high levels of IFN-γ and TNF-α (characteristic of Th1 immune responses). Th2 cytokines, IL-4 and IL-5, were detected in very low levels with no difference from the control group. Our group has previously performed other vaccine trials and the majority of *S. mansoni* antigens tested as vaccine candidates that conferred partial protection against cercariae challenge induced a Th1-type immune response [15], [32], [41], [42]. In the case of vaccination with *Sm*Synt, the shift to Th1 occurred after the third immunization and was maintained after the challenge with cercariae, which helped to control schistosomiasis infection. It has been shown that IFN-γ promotes the recruitment and activation of macrophages to kill worms [49]. Further, IFN-γ knock-out mice (or mice treated with specific anti-IFN-γ antibodies to deplete their IFN-γ levels) are unable to protect against schistosome infection [50], [51]. Both IFN-γ and TNF-α increase the levels of nitric oxide produced by recruited macrophages, which is also related to a protective immune response against *S. mansoni* [51]. With this description of the immune profile of r*Sm*Synt-vaccinated mice, we next investigated whether this molecule could induce protection in a murine model of *S. mansoni* infection. Two independent experiments were performed and, in both, the vaccine conferred partial protection resulting in 30–37% reduction in worm burden. It also ameliorated liver pathology, as shown by a 38–42% reduction in the number, and a 35–37% reduction in the area, of granulomas. These liver pathology results might be related to the elevated levels of IL-10 detected in the supernatant of cultured, stimulated splenocytes from r*Sm*Synt-immunized mice. High levels of IL-10 were detected in the end of immunization schedule and also after the infection with cercariae. Based on these results, our hypothesis is that this cytokine might play a role in the regulation of Th2 responses, reducing the inflammation in liver pathology [52].

In summary, here, we demonstrate that *SmSynt* gene is mainly expressed during the intravascular life stages and it is located in the gastrodermis of adult worms, suggesting its possible role in the blood feeding process. Knocking-down expression of *SmSynt* has no noticeable impact on the parasites *in vitro*. However, vaccination of mice with r*Sm*Synt generates a Th-1 immune response which partially protects them against challenge schistosome infection and leads to reduced liver pathology. This outcome makes *Sm*Synt a candidate for further development as an anti-schistosome vaccine, perhaps as part of a multiple, protective-antigen cocktail.
